# Peptide Interference with APP and Tau Association: Relevance to Alzheimer’s Disease Amelioration

**DOI:** 10.3390/ijms21093270

**Published:** 2020-05-05

**Authors:** Ruth Maron, Gad Armony, Michael Tsoory, Meir Wilchek, Dan Frenkel, Ruth Arnon

**Affiliations:** 1 Department of Immunology, Weizmann Institute of Science, Rehovot 76100, Israel; ruth.maron@weizmann.ac.il; 2 Department of Structural Biology, Weizmann Institute of Science, Rehovot 76100, Israel; gadi.armony@gmail.com; 3 Department of Veterinary Resources, Weizmann Institute of Science, Rehovot 76100, Israel; michael.tsoory@weizmann.ac.il; 4 Department of Biomolecular Science, Weizmann Institute of Science, Rehovot 76100, Israel; Meir.wilchek@weizmann.ac.il; 5 Department of Neurobiology, School of Neurobiology, Biochemistry and Biophysics, The George S. Wise Faculty of Life Sciences, Sagol School of Neuroscience Tel Aviv University, Tel Aviv 6997801, Israel; dfrenkel@tauex.tau.ac.il

**Keywords:** APP and Tau protein, synthetic peptides, Alzheimer’s disease, cognitive function, amyloid plaques

## Abstract

The two major proteins involved in Alzheimer’s disease (AD) are the amyloid precursor protein (APP) and Tau. Here, we demonstrate that these two proteins can bind to each other. Four possible peptides APP1 (390–412), APP2 (713–730), Tau1 (19–34) and Tau2 (331–348), were predicted to be involved in this interaction, with actual binding confirmed for APP1 and Tau1. In vivo studies were performed in an Alzheimer Disease animal model—APP double transgenic (Tg) 5xFAD—as well as in 5xFAD crossed with Tau transgenic 5xFADXTau (FT), which exhibit declined cognitive reduction at four months of age. Nasal administration of APP1 and Tau1 mixture, three times a week for four or five months, reduced amyloid plaque burden as well as the level of soluble Aβ 1–42 in the brain. The treatment prevented the deterioration of cognitive functions when initiated at the age of three months, before cognitive deficiency was evident, and also at the age of six months, when such deficiencies are already observed, leading to a full regain of cognitive function.

## 1. Introduction

Peptides serve as important research tools in elucidating protein functions. They are also used to verify and identify protein–protein interactions [1] and their roles in controlling cellular functions and processes that occur in living organisms [2]. Peptides and polypeptides have been investigated in our laboratory for decades. They were instrumental in establishing the chemical basis of protein antigenicity [3] for preparing the first synthetic polypeptide antigen [4], for the first synthetic antigen leading to an immune response against a native protein [5] and for developing an effective drug to treat multiple sclerosis [6,7]. Peptides have also served as the basis for the first antiviral synthetic vaccine [8], which has led to the concept of epitope-based antiviral and anti-influenza vaccines [9,10] established in our laboratory. 

Alzheimer’s disease (AD) is the most common form of age-associated neurodegenerative disorder, clinically characterized by a decline in cognitive function or dementia [11]. Pathologically, it is defined by the accumulation of extracellular beta-amyloid (Aβ) plaques and intracellular neurofibrillary tangles (NFTs). The Aβ plaques are comprised of fragments of 40 or 42 amino acid residues produced by proteolytic cleavage of the APP protein [12], while NFTs are composed of hyperphosphorylated Tau protein [13]. While it was suggested that phosphorylation of Tau might be important for its activity, other sites of phosphorylation are linked to AD pathology. The causes or mechanisms of these plaque formations and tangles are not yet well understood, but basically it is considered a protein-misfolding process that leads to the disease. The short peptide segments of the Aβ plaques were demonstrated to be abnormally folded [14]. Tau is a microtubule-associated protein expressed in the neurons that normally acts to stabilize the microtubules in the cell cytoskeleton, and is regulated by phosphorylation. Hyperphosphorylated Tau is associated with misfolding and aggregation, and correlates with impaired cognitive functions [13]. Consequently, regarding the mechanism of AD, there are two schools of thought as to whether APP (amyloid beta) or Tau are the causative factor in the disease [15]. However, ongoing work focusing on single target therapies was disappointing and therefore, dual amyloid and Tau targeting approaches are possibly more effective [16].

The hypothesis behind the present study is that the interaction between APP and Tau is important in the induction and/or progression of the disease. An indication for a direct interaction of Tau protein with APP was already seen in 1995 [17]. It was shown that the APP peptide identified in this interaction spontaneously formed fibrils in vitro; furthermore, in the presence of Tau, APP generated dense fibrillary assemblies containing both molecules [18]. Nevertheless, no indication was provided in these studies to the region in the Tau molecule involved in binding to APP.

The present study focused on interactions between purified recombinant molecules of APP and Tau. We demonstrate here that these two molecules are indeed capable of binding to each other, probably via a single peptide region in each protein. We have predicted the respective two peptides that are involved in this binding and demonstrated that their synthetic versions are capable of forming a complex. We further demonstrated that nasal administration of a mixture of these two peptides reduces brain plaque formation, decreases soluble Aβ 1–42 (amyloid brain load) in homogenized brains, and significantly improves the cognitive function in an AD animal model.

## 2. Results

### 2.1. Verification of APP and Tau Protein Binding and Crosslinking of the Proteins

**Figure 1:**
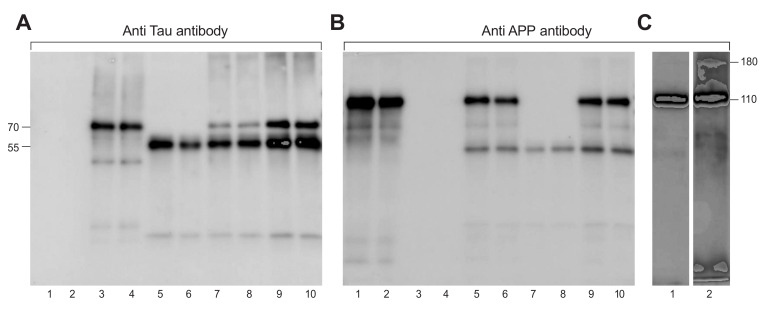
Western blot of APP and Tau protein binding and crosslinking of the proteins. Samples loaded in duplicates in blots A and B. In blots (**A**,**B**) Lanes 1 and 2 are loaded with 50ng APP protein/lane. Lanes 3 and 4 are loaded with 50ng/lane Tau protein. APP-His protein bound to protein A/G beads, previously coupled with anti-His antibodies are loaded on lanes 5 and 6. Tau protein bound to protein A beads coupled with anti-His antibodies are loaded in lanes 7 and 8. In the last two lanes, 9 and 10 of A and B, a mixture of APP and Tau proteins was loaded. The mixture was performed by binding APP-His protein to protein A/G beads, previously coupled with anti-His antibodies, and then Tau protein was added (Materials and Methods section). Membrane (**A**) was developed with anti-Tau antibody and was overexposed to enable detection of any non-specific binding of Tau (70 kD), also causing overexposure of the IgG band (55 kD). The faint band of Tau seen in Figure 1A, lanes 7 and 8 is non-specific as Tau was bound to a negative control protein (45 kD bacterial protein-His). Therefore, we concluded that the strong tau band seen in lanes 9 and 10 was specific binding of Tau to APP. Membrane (**B**) was developed with anti- Aβ antibody (6E10) and shows the 110 kD band of APP protein. Membrane (**C**) was loaded with APP protein on lane 1 and with a mixture of APP and Tau, crosslinked on lane 2 (Materials and Methods section).

The first step was to determine if the APP protein binds to Tau. As seen in Figure 1A, anti-Tau antibodies labelled Tau protein alone (70 kD, lane 3 and 4) and Tau bound to APP-His beads (lanes 9 and 10), on a Western blot. An additional band (55 kD) of immunoglobulin was seen in the samples that were bound to beads containing anti-His antibodies (lanes 5 to 10). Figure 1B has the identical loading plan as in Figure 1A, but the blot was incubated with anti-Aβ antibodies (6E10) which bound to the APP protein. Here, APP, APP-His protein bound to beads or Tau bound to APP-His beads demonstrated 110 kD band (lanes 1, 2, 5, 6, 9, 10) which is the MW of APP. Thus, Figure 1A,B confirm that the two proteins which are the main components contributing to AD bind to each other. 

As for a negative control, we used an inside control by using our proteins APP with anti-Tau antibodies on blot 1A lanes 1 and 2 and Tau protein with anti-APP antibodies on blot 1B lanes 3 and 4. Membrane (C) was loaded with APP protein on lane 1 and with a mixture of APP and Tau, crosslinked on lane 2 (Materials and Methods section). Blot (C) was developed with anti-Aβ antibody. Depicted in lane 1 APP protein (110 kD), Lane 2, which was loaded with the crosslinked APP and Tau proteins, shows the 110 kD band of APP as well as a 180 kD band, which is the expected molecular weight of APP and Tau together. Membrane (C) was overexposed when developed to be able to see the 180 kD crosslinked band and therefore, the APP bands are very dark.

We then chemically crosslinked the proteins using BS3-H12/D12, and searched for a crosslinked product of both proteins, which would indicate that the proteins bind to each other. As shown in Figure 1C, the anti-APP antibodies labelled the band of APP protein (lane 1 and 2), as well as a 180 kD band representing the crosslinked APP (110 kD) and Tau (70 kD) proteins (lane 2). 

### 2.2. Prediction of the APP and Tau Peptides Involved in the Protein Binding

Using the UNIPROT [19] and BLASTP [20] programs, we identified areas in both the APP and Tau protein as possible areas of interaction between the two proteins. Likely candidates were the locations on APP that align with both GSK3 and ApoE3, and the microtubule-associated protein region, and/or the N-terminal region of Tau. Upon analysis of the crosslinked material (Figure 1C, lane 2), which was processed for LC-MS/MS, only one crosslink was identified between APP and Tau, between lysine 370 on APP and lysine 387 on Tau. The crosslinked lysine for APP resides very close to the accordingly predicted APP1 (390–412) peptide, as can be seen by a crystal structure of the region [21]. We also tested, as a control, the APP peptide previously reported to bind to Tau [18], namely APP2 (residues 713–730). As for Tau, we selected the peptide Tau1 (residues 19–34) which is in the N-terminal end of Tau protein, since phospho-Tyr-hTau located in the N-terminal was reported to accompany AD progression and Tauopathy [22]. We also selected peptide Tau2 (residues 331–348) from the microtubule area of Tau protein, which is proximal to the crosslinked lysine 387. All four peptides were synthesized and used for the in vitro and in vivo experiments. The peptide sequences are: APP1 (HFQKAKERLEAKHRERMSQVMRE); APP2 (ATVIVITLVMLKKKQYTS); Tau1 (GLGDRKDQGGYTMHQD); Tau2 (KPGGGQVEVKSEKLDFKD).

### 2.3. In Vitro Assessment of the APP and Tau Protein Peptides and Their Binding

**Figure 2:**
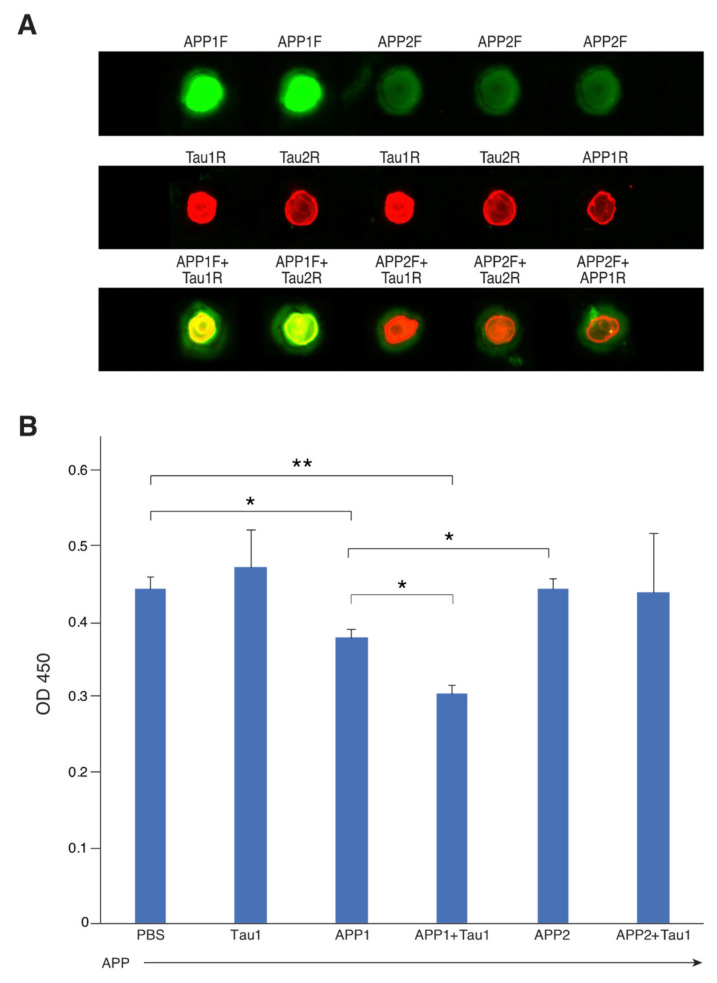
Confirmation of APP and Tau peptide binding by visualization and ELISA. (**A**) Visualization of APP and Tau peptide binding labelled by fluorescein (F) or rhodamine (R). Single labelled peptides were loaded on nitrocellulose membrane [upper row (F) and middle row (R)]. Mixtures were loaded (lower row) from left to right: APP1F + Tau1R, APP1F + Tau2R, APP2F + Tau1R and APP2F + Tau2R. Last sample was a control of APP2F + APP1R. As can be seen the only two peptides that bound to each other were APP1F and Tau1R as the color mix combination of green and red resulted in yellow (except for a faint yellow color for the combination of APP1F + Tau2R). The dot blot seen in the figure is a 1 to 1 representation of the dot seen on the nitro-cellular blot. (**B**) Peptide inhibition of Tau and APP protein binding. An ELISA plate was coated with Tau protein (1 µg/ml) overnight (ON) at 4 °C. PBS, single peptides (1 µg/ml, 50 µl/well) or their combinations (0.5 + 0.5 µg /ml) were incubated ON at 4 °C as well. The next day, the plate was washed and blocked with PBS + 3% BSA for two hours at RT. The plate was washed again with PBS. Single peptides or their combinations were added to the coated plate for four hours at RT. The plate was washed and APP protein was added to all wells for ON incubation at 4 °C. The plate was then washed and anti-Aβ was added, to test the ability of the different peptides to affect APP protein binding to the Tau coated plate. The plate was developed with anti-mouse HRP (materials and methods section). Samples were in triplicates. Significance labelled as *p* = 0.05*, *p* = 0.008**.

All four candidate peptides were labelled with fluorescein. APP1, Tau1 and Tau2 were also labelled with rhodamine for visualization of peptide binding. The dot-blot assay (Figure 2A) shows that the only two peptides that bound to each other are APP1F + Tau1R, as seen by the yellow color. All other combinations resulted in separate red and green dots (except for a faint yellow color for the combination of APP1F and Tau2R).

The next step was to determine (by ELISA) which of the single peptides or their mixtures inhibits the binding of APP and Tau. Figure 2B represents the results of one experiment, run in triplicates, out of three repeated experiments. We used only one concentration of peptide that was found to be beneficial in all of our other measurements described in the paper, Figure 2B shows that APP–Tau binding is not inhibited by Tau1 or APP2. A partial inhibition was seen with APP1. However, the combination of APP1 and Tau1, which was the only combination shown to bind by the dot blot (Figure 2A), had a more significant inhibitory effect on the binding of the two proteins. 

### 2.4. In Vivo Treatment of 5xFADXTau (FT) Mice or 5xFAD with APP1 and Tau1 Mixture and Its Effect on Cognition, Plaques and Soluble Brain Aß 1–42 Levels

#### 2.4.1. Outline of Experimental Process

**Figure 3:**
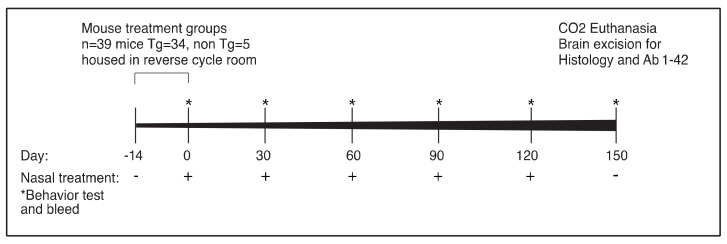
In order to test our peptides in vivo, we moved our experimental mice to a reverse cycle room 2 weeks prior to the beginning of treatments and tests. The animal model used was 5xFAD APP Tg, or 5xFAD mice crossed with Tau Tg mice 5xFADXTau (FT). Mice were tested before the treatment began (behavior tests). Mice were treated with either APP + 1 or Tau1 mixture or PBS as the control was given 3 times per week. Once a month, during the experiment, mice were tested for cognitive function. The assessments included the Y-maze test assessing spatial recognition memory, as a hallmark of cognition function [23] and the open field (OF) test, an established anxiety and basic motor functions test [24], to control for confounding factors that may affect the behavior in the Y-maze. The experiment ended by euthanizing the mice and excision of their brains. One hemisphere was prepared for histology and the other was frozen in −70 °C for processing to test Aβ 1-42 content.

The in vivo research design employed in the study is illustrated in Figure 3.

#### 2.4.2. Cognitive Functions 

The FT or 5xFAD mice used show cognitive impairments at the age of four months. Behavioral assessments were conducted before starting the treatment, at the age of either three months (before cognitive impairment) or six months (after significant impairment was evident), and then once a month during the treatment period, for a total of four or five assessment sessions. The assessments included the Y-maze test, assessing the spatial recognition memory, a hallmark of cognition functions, as well as the open field (OF) test, an established anxiety and basic motor functions test, controlling confounding factors that may affect behavior in the Y-maze. Control mice were 5xFAD or FT mice treated with PBS, or non-Tg littermates treated with the peptide mixture. At the end of the experiment, the mice were sacrificed and their brains excised. One half of the brain was prepared for histology and one half was frozen at −70 °C for soluble Aβ 1-42 measurement.

**Figure 4:**
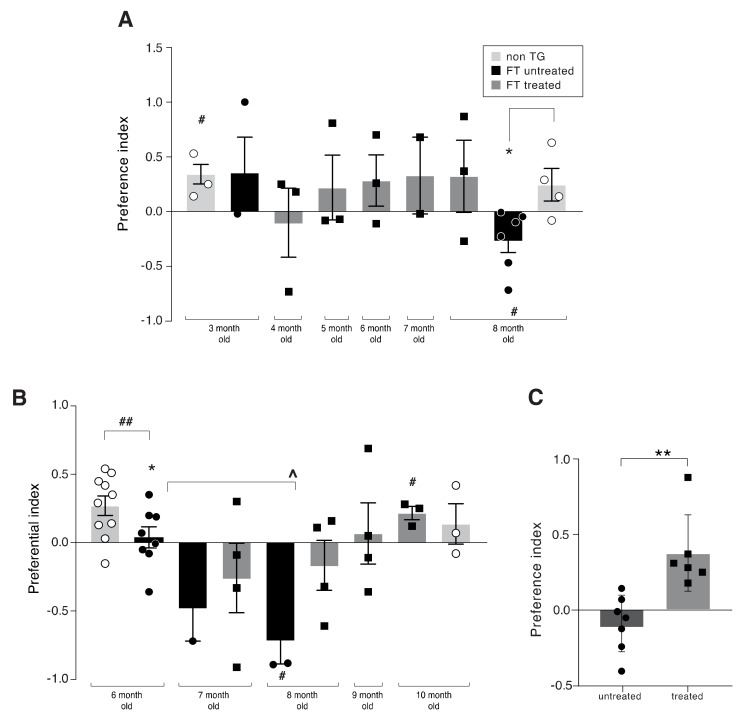
In vivo, monthly behavior follow-up of 5xFADXTau (FT) mice treated with a mixture of APP1 and Tau1 peptides versus control PBS treated mice. (**A**) “Novel” arm differential preference index among control (non-transgenic) and transgenic FT mice, treated and non-treated (PBS treated), between the age of three to eight months. At the age of three months, only non-Tg control mice exhibited a significant preference to the “Novel” arm ((# *p* = 0.016). The benefits of the treatment were evident at the end of the five months course. At the age of eight months, only non-treated FT mice exhibited significant poor Y-maze performance (# *p* = 0.035) and differed significantly from non-Tg control mice (*p = 0.048). Each treatment group included *n*= 3–4 mice. (**B**) “Novel” arm differential preference index among control (non-transgenic) and transgenic FT mice, treated and non-treated (PBS treated), between the age of 6 to 10 months. At the age of six months, only non-Tg control mice exhibited a significant preference for the “Novel” arm in the Y-maze (## *p* = 0.002), while non-treated FT mice performed significantly worse than non-Tg controls (* *p* = 0.022). A significant (*p* = 0.022) age associated cognitive decline was noted among non-treated FT between 6–8 months, where eight months old non-treated FT mice performed significantly worse than six months old non-treated FT mice (^ *p* = 0.029) and exhibited a significant negative differential preference to the “Novel” arm (# *p* = 0.028). The benefits of the treatment were evident at the end of the treatment course at the age of 10 months, when only treated FT mice exhibited a significant differential preference to the “Novel” arm (# *p* = 0.024). Each treatment group included 3–4 mice. (**C**) Preferential index of untreated versus treated FT mice. FT Tg mice nasally treated with APP1 + Tau1 mixture versus PBS control as described in Figure 2B. Treatment continued between the age of 6–10 months and tested by Y-maze at the age of 10 months. Control treated mice did not recognize the “Novel” arm, while the APP1 + Tau1 treated mice had a good cognitive score as measured by the preferential index of the “Novel” arm (*n* = 6–7 mice, ** *p* = 0.0029).

Figure 4A depicts the cognitive functions, assessed in the Y-maze, of control (non-transgenic) and transgenic FT mice, treated and non-treated, compared between the ages of three to eight months. At the age of three months, the performance of the transgenic and control groups were similar, exhibiting preference to the “Novel” arm (Statistical significance, “0” [t_(3)_ = 3.824; *p* (one-sided) = 0.016], was noticed only by the non-Tg control). The number of mice per group in the in vivo studies is small due to logistic shortage in the number of the double transgenic FT mice. However, the number we used still enabled us to have statistical significance, suggesting a strong effect of the therapeutic intervention described here.

The benefit of the treatment was evident at the end of the treatment course at eight months, when only non-treated FT mice exhibited significantly poor Y-maze performance. In addition, although ANOVA did not indicate a significant difference between the groups at eight months [F_(2)_ = 3.658; *p* = 0.064], Dunnett’s post hoc comparisons (one-sided) indicated that untreated FT differed significantly from non-Tg control mice (*p* = 0.048), whereas treated FT mice did not differ from non-Tg control mice (*p* = 0.471).

Figure 4B illustrates the cognitive functions, assessed in the Y-maze, of control (non-transgenic) and FT mice, treated and non-treated, that were compared between the ages of 6 to 10 months. At six months, only non-Tg control mice exhibited a significant preference to the “Novel” arm in the Y-maze [t_(9)_ = 3.780; *p* (one-sided) = 0.002]. Untreated FT mice performed significantly worse than non-Tg controls [t_(16)_ = 2.198; *p* (one-sided) = 0.022] and did not exhibit a significant differential preference to the “Novel” arm [t_(7)_ = 0.508; *p* (one-sided) = 0.314]. During the experiment period the untreated FT mice deteriorated rapidly, whereas the peptides-treated mice showed initial deterioration followed by regain of cognitive function.

Kruskal–Wallis analysis indicated a significant age-associated cognitive decline only among non-treated FT mice between six to eight months. Dunn’s post hoc comparisons indicated that eight months old non-treated FT mice performed significantly worse than six months old non-treated FT mice (*p* = 0.029). In addition, eight months old non-treated FT mice exhibited a significant negative differential preference to the “Novel” arm, i.e., <“0”. ANOVA did not indicate significant differences in FT treated mice over four months of treatment, but the benefits of treatment were evident at the end of the treatment course. At the age of 10 months, only treated FT mice exhibited a significant (*p* = 0.024) differential preference to the “Novel” arm. Figure 4C summarizes the cognitive performance (preferential index) obtained from 10 months old FT mice nasally treated for four months with the APP1 and Tau1 peptide mixture, indicating the significant effect of the treatment. Additional assessments evaluated the effects of the treatment on both basic locomotor functions and anxiety using the open field (OF) test. The results of the open field (OF) tests did not show any differences in anxiety throughout the five months course of treatment between control non-Tg and treated FT or 5xFAD mice. In addition, the groups did not differ substantially in their motor functions. Hence, the effect of treatment on cognition was confounded by locomotor functions or anxiety.

**Figure 5:**
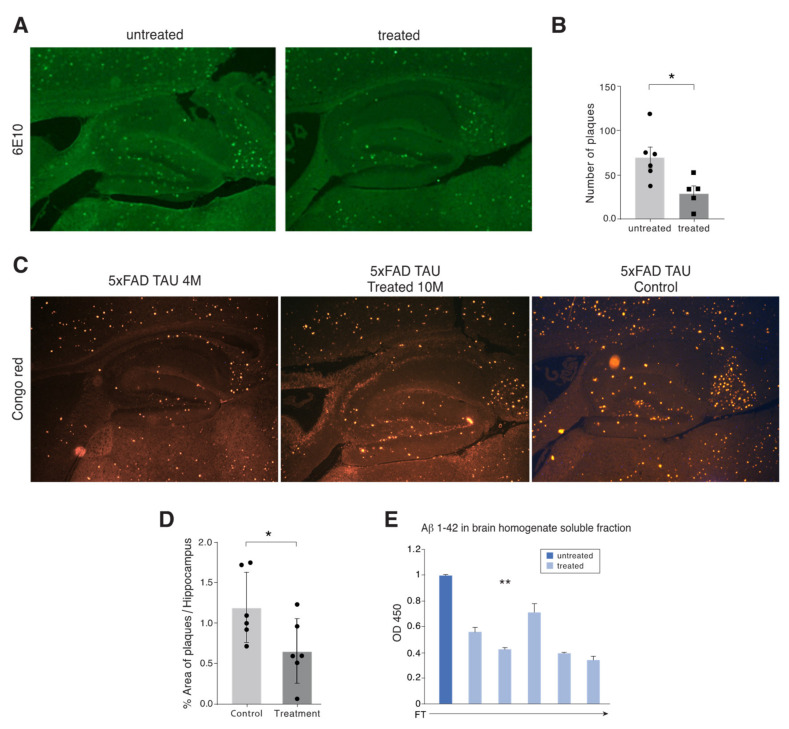
APP1 + Tau1 mixture nasally treated FT mice show reduced % plaque area, reduced brain amyloid load, in correlation with improved cognition. (**A**) Histological images of sagittal sections stained with anti-Aβ 6E10 antibodies from brains of FT Tg mice non-treated versus nasally treated with APP1 + Tau1 mixture. Treatment was given between the ages of 6 to 10 months. Histological images stained with 6E10 antibodies of 10 months old FT non-treated mice have an accumulation of a large number of Aβ plaques (left panel). However, 10 months old APP1 + Tau1 mixture treated mice have a significantly smaller number of plaques (right panel). Original magnification ×4. (**B**) Quantification of number of plaques in hippocampal sections from 5xFADXTau (FT) mice with or without treatment determined by 6E10 antibody staining (*n* = 5–6 mice, * *p* = 0.02). Original magnification ×4. (**C**) Histological images of sections, stained with Congo red, from brains of FT Tg mice nasally treated with APP1 + Tau1 mixture versus PBS control. Treatment was given between the ages of 6 to 10 months. Histological images of sections stained with Congo red of four months old FT non-treated mice already have some accumulation of Aβ plaques (left panel). However, 10 months old PBS treated mice had a much larger % area of plaques (right panel). In contrast, 10 months old APP1 + Tau1 mixture treated mice had a much smaller accumulation of plaque area (middle panel). (**D**) Quantification of % plaque area of hippocampal sections from 5xFADXTau (FT) mice with or without treatment determined by Congo red staining (*n* = 6 mice, *p = 0.049). (**E**) ELISA assessment of soluble Aβ (1–42) in FT mice. The right hemisphere of each mouse in the treatment group was homogenized with PBS containing protease inhibitor and centrifuged at 40.0009g for 40 min to quantify soluble Aβ levels found in the supernatant. Levels of Aβ (1–42) in brain samples were assessed by an ELISA kit for high sensitivity human amyloid beta-42. Control PBS treated mice (n = 2) versus five single mice treated with APP1 + Tau1 n = 5 (** *p* = 0.006). ELISA samples were done in triplicate.

To address the issue of an association between amyloid plaques and cognitive decline, we measured both insoluble Aβ represented by plaques and soluble Aβ in the brain. The mice were sacrificed at the end of the experiment and their brains excised to quantify the percentage of brain area in which plaques were present. Sagittal brain sections were stained with either anti-Aβ 6E10 antibody (Figure 5A) or Congo red dye (Figure 5C). Quantification of Aβ depositions was done for the hippocampus area in a blinded fashion using Imaging Research software from National Institutes of Health in an unbiased stereological approach. For Congo red, the results presented are percentage of area of congophilic staining versus total area of measured hippocampus. For 6E10 staining, the results are presented as number of positive staining per total hippocampus region (Figure 5B), indicating significant reduction as the result of treatment. At the age of ten months, the % plaque area in FT mice treated with the APP1 + Tau1 peptide mixture was significantly reduced (Figure 5C, middle panel), a substantial reduction of more than 45% as compared with the non-treated FT mice. In addition, staining sections of brains with 6E10 anti-Aβ antibodies of untreated 10 months old mice revealed a significantly higher number of plaques than the same age treated mice (Figure 5A,B). 

**Figure 6:**
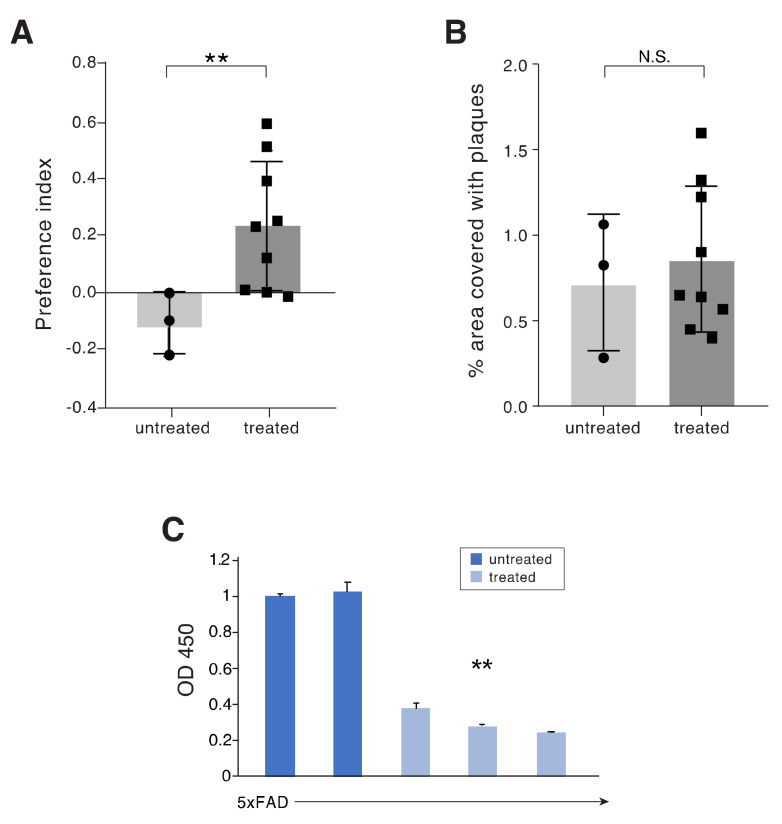
5xFAD nasally treated mice with APP1 + Tau1 peptide mixture show improved cognition in correlation with reduced brain amyloid Aβ (1–42) load. (**A**) Preferential index of control, PBS treated versus mixture treated 5xFAD mice. Treatment continued between the ages of three to eight months and tested by Y-maze at the age of eight months (as described in Figure 4A). Control PBS treated mice did not recognize the “Novel” arm, while the mixture treated mice had a good cognitive score as measured by the preferential index of the “Novel” arm. (*n* = 3 control treated *n* = 9 peptide treated mice *p* = 0.0344). (**B**) Quantification of % plaque area of hippocampal sections from 5xFAD mice control treatment (PBS) versus peptide treated, determined by Congo red staining (*n* = 3 control mice, treated = 9 mice, *p* = N.S.). (**C**) ELISA assessment of soluble Aβ (1–42) in brains from 5xFAD mice. Procedure done as described in Figure 5E. Levels of Aβ (1–42) in brain samples were assessed by an ELISA kit for high sensitivity human amyloid beta-42. Control PBS treated mice (*n* = 2) versus *n* = 3 mice treated with APP1 + Tau1 peptides (** *p* = 0.0015). ELISA samples were done in triplicate.

Another experiment was performed in 5xFAD mice treated with APP1 + Tau1 peptide mixture for five months, starting at the age of three months. The majority of the treated mice at eight months of age showed, as did the FT mice, an increased preferential index as compared to PBS treated 5xFAD mice, indicating the significance of the treatment (Figure 6A, *p* = 0.0344). However, no reduction in the plaque load was observed in brains of the treated 5xFAD APP mice (Figure 6B), and hence no correlation with cognitive function. 

Recently, the soluble Aβ has become the focus of AD research and was suggested to contribute to AD development [25]. We therefore assessed by ELISA the content of soluble Aß 1-42 in the brains. Soluble Aβ 1–42 was significantly lower in the brains of the peptide treated mice, in both FT and 5xFAD, than in the control (PBS) treated mice (Figure 5E and Figure 6C, *p* = 0.006). The data presented in Figure 6C are each of an individual mouse, two 5xFAD Tg PBS treated and three 5xFAD Tg treated with APP1 +Tau1 mixture. The results of each mouse is an average of a triplicate ELISA reading.

The results of the open field (OF) tests did not show any differences in anxiety throughout the five month course of treatment between control non-Tg and treated FT or 5xFAD mice. In addition, the groups did not differ substantially in their motor functions. Hence, the effect of treatment on cognition was not confounded by locomotor functions or anxiety. 

## 3. Discussion

Proteins, which are involved in a variety of structural and physiological functions, constitute an integral component of cells and their function [2]. Diverse cellular signaling processes and crosstalk involve protein-protein interactions [26]. Disruption of such interactions can have significant consequences in various biological processes related to health and disease. Alzheimer’s disease is a multifaceted disorder that is associated with several protein-protein interactions. These include the Aβ self-aggregation resulting in amyloid plaques, as well as self-aggregates of Tau, resulting in neurofibrillary tangles. Several other protein-protein aggregates, including that of APP and β-secretase 1 (BACE 1), and those involving cellular prion proteins, phosphoprotein phosphatase 2A and Mint 2, have also been reported as having a role in AD progression [27]. In the present study, we explored the possible interaction between APP and Tau, and its role in the disease process. Once demonstrating that such an interaction (binding) exists, we studied the effects of its inhibition. To predict the molecular sites through which these two proteins may interact with each other, we initially resorted to a computer-assisted methodology using the BLASTP program [19], relying on primary structure, since only partial information exists for the three dimensional structure of APP [28], and lately, the structure of Tau filaments is available by cryo-Electro-Microscopy analysis which allowed atomic characterization of amyloid filaments from patient-derived brains. Filament cores are made of two identical protofilaments comprising residues 306–378 of Tau, which adopt a combined cross-beta/beta-helix structure and define the seed for Tau aggregation [29]. 

A more direct, experimental approach was the crosslinking of the two proteins, followed by enzymatic digestion of the conjugate and identifying the fragment(s) that consist of sequences from both proteins. 

The analysis we performed led to region 389–411 of APP as a possible peptide candidate. The relevance of the peptide with this sequence that was synthesized (denoted APP1) was confirmed by the crosslinking experiment, in which the linking sequence LPTTAAS was found close to the APP1 peptide. Another APP peptide tested, denoted APP2, is the region 713–730 which was previously reported to bind to Tau protein [18]. 

As for Tau, we also tested two hypothetical peptides: The peptide denoted Tau1 (residues 19–34) was chosen as there is some evidence that the N-terminal domain of Tau (18–28) acts as a binding motif for End Binding proteins and could facilitate Tau secretion to the extra cellular space [30], or bind to extracellular APP. It was also reported that phopspho-Tyr18-hTau, in the N-terminal domain, was found to accompany disease progression of Alzheimer’s disease and Tauopathy [22]. The other Tau peptide denoted Tau2 (residues 331–348) was selected from the microtubule associated protein found in the C-terminal area of Tau protein. The identification of the sequence KIETH, in our crosslinking experiments, close to the sequence of Tau2, supported its relevance. When the four selected peptides were tested by direct binding experiments as well as for their ability to inhibit APP binding to Tau, only the mixture APP1 + Tau1 was found to be relevant. Hence, all in vivo studies have been performed with a mixture/complex of these two peptides. It should be emphasized that above the four peptides predicted and used by us, other peptides of the APP and Tau proteins could also be effective to ameliorate amyloid pathology, as described in [31,32]. 

The major animal model used in this study was the 5xFADXTau (FT) Tg mice, which is a cross between 5xFAD mice [33] carrying three familial APP mutations and two PSEN1 mutations, and Tau Tg mice with two mutations K257T/P301S (double mutant DM) [34]. The phosphorylation in Tau Tg mice that was used in our FT mouse model was linked to pathology and cognitive impairment. The rationale for using this model, with human mutations in both APP and Tau, is to allow an effect of a complex of these two proteins, if such exists. The 5xFAD Tg mice show many familial AD-related phenotypes and have a relatively early and aggressive presentation. Amyloid plaques are frequently detected in the 5xFAD mice as early as three months of age [33]. The mice display a range of cognitive deficits, including memory deficits by six months of age [35]. Hence, this model was also used in the present study. Of note, depletion of Tau in 5xFAD mice resulted in reduced brain amyloid load, both soluble and insoluble, as well as neuronal death amelioration, suggesting a potential direct link between APP and Tau. It was also previously reported that there is endogenous elevation in Tau phosphorylation at Ser396 in 5xFAD mice [36]. This finding suggests a strong link between elevations in APP levels and increased Tau phosphorylation prior to memory impairment in this model. 

The double transgenic FT mice used in the present study express, in addition to the mutations in 5xFAD, additional mutations in Tau and were expected to exhibit an aggressive phenotype. Indeed, in the group of untreated control FT mice, a deficit in cognitive function was observed at four months of age, compared to non-transgenic mice of the same age. We anticipated that the peptide complex stemming from the two proteins might affect aggregates of either APP or Tau, or of both proteins, if such exist.

In FT mice treated with the APP1 + Tau1 peptide mixture, an initial reduction in cognitive function was observed, followed by a gradual reversal of this effect, resulting in a full recovery of cognitive function, comparable to control non-transgenic mice of the same age at the end of the treatment course. Furthermore, plaque formation representing insoluble Aβ, as well as soluble **Aβ** 1-42 load in their brains, was drastically reduced in the treated mice compared to the PBS treated controls. Of note, our results suggest a link between improvement in cognition in treated Tau APP mice versus control to reduction of Beta amyloid plaques. In line with these results, it was previously shown that administration of human paired helical filaments (PHF), composed of abnormally phosphorylated and aggregated microtubule-associated Tau protein, to brains of 5xFAD mice resulted in increased aggregation of endogenous murine Tau as compared to wild-type, suggesting a link between APP and Tau aggregation [37]. 

The present results could have a possible implication towards drug development for the treatment of AD. Were the positive effect on cognitive function observed only in FT mice exhibiting mutations in both APP and Tau, one could argue that such mutations are not necessarily seen in AD patients. The positive effect of the peptides mixture on the cognitive functions in the 5xFAD animal model refutes this argument. A significant difference between the two animal models is that in the FT mice, the peptide treatment reduced drastically the plaque accumulation in the brain, an effect that was not observed in the 5xFAD mice. However, accumulation of soluble Aβ 1-42 was reduced in brains of both models.

## 4. Materials and Methods

### 4.1. Proteins, Chemicals and Antibodies

Recombinant human APP770, (BLG843201) Recombinant human APP770-His tag, Recombinant human Tau-441 (2N4R) (BLG6842501) and purified anti-amyloid 1-16 6E10 (RRID:RB_2564653) were all purchased from Bio-Legend, San Diego, CA 92121 USA. Anti-Tau antibodies (anti-Tau pantropic, at-5004) were purchased from MBL, Ottawa. IL 61350 USA. Anti-mouse HRP was from Jackson Immunoresearch, Westgrove, PA, USA, 19390 (Cat#715-035-151). Mouse anti-glyceraldehyde 3-phosphate dehydrogenase antibody (GAPDH) Cat#MAB374 purchased from Merck-Millipore, MA., USA Fluorescein (Cat#F7250) or rhodamine (Cat#R-1755) isothiocyanate and Congo red were purchased from Sigma-Aldrich Rehovot, Israel, 76100. Bissulfosuccinimidyl suberate crosslinker (BS3-H12/D12 Cat#00155) was from Creative Molecules Inc. Protein A/G agarose beads (Cat#SC2001) were from Santa Cruz, California USA. Human amyloid 1–42 high sensitivity ELISA (Cat#EZHS42) was from Merck, Albany, NY, USA.

Chemicals and antibodies were purchased between 2016–2019. 

### 4.2. Mouse Lines

The following mice were used: 5xFAD male and female double transgenic mice (Tg6799 line APP/PS1), (Jackson, JAX: 032284), co-expressing the human amyloid precursor protein carrying five familial Alzheimer’s disease mutations: the Swedish, Florida and London mutations and two mutations of the human presenilin-1 [33]. The DM hTau transgenic mouse line expressing two mutations was obtained from Hana Rosenmann [34]. All mouse lines were maintained on a C57Bl/6 background (Jackson, RRID:IMSR_JAX:000664). We used heterozygous male 5xFAD Tg mice for crossing with female DM hTau heterozygous Tg mice to produce double Tg mice 5xFADXTau (FT). Genotyping was performed by PCR amplification of tail DNA, as previously described [38]. The mice were housed in individually ventilated cages (no more than 5 mice per cage) in a temperature-controlled facility with a 12-h light/dark cycle. All animal care and experimental use were in accordance with the Weizmann Institute of Science guidelines and were approved by the Weizmann Animal Care Committee IACUC #34030217-3 (29 November 2017–29 November 2019). Animal weight was 20–25 g and mice were given food and water ad libitum.

### 4.3. Western Blot Analysis of APP and Tau Protein Binding

Western blot was performed as previously described [39]. Loaded samples were prepared as follows: Anti-His antibodies (1–2 µg) were bound to protein A beads (10 µL) in a microfuge for one hour at room temperature (RT). Beads were washed with phosphate-buffered saline (PBS) containing only 50mM NaCl (which was used all along for washes), centrifuged and supernatant (sup) aspirated. APP-His (1 µg) was added and incubated for one hour at RT and the complex was washed again, centrifuged and then, sup aspirated. Tau protein (1 µg) was added for a one hour incubation at RT. As a negative control, Tau protein was added to protein A beads and incubated overnight (ON) at 4 °C. Before loading, samples were centrifuged, sup aspirated and 80 µl PBS + 20 µl loading buffer (5×) was added. Samples were boiled, centrifuged and 20 µL of each sample was loaded as well as 50 ng in 20 µL of either APP protein or Tau protein. For electrophoretic separations, 10% polyacrylamide gels were used and transferred to nitrocellulose membranes. The membranes were blocked with PBS 5% bovine serum albumin (BSA) for 1 h, then washed with 0.05% Tween in tris-buffered saline (TBST) and reacted with either anti-Aβ antibodies (6E10 1:250 dilution) or anti-Tau antibodies (anti-Tau, 1:2500 dilution) ON at 4 °C. The membranes were washed three times with PBS-Tween (0.05%) and reacted with secondary anti-mouse HRP (1:10,000) for 1h at RT. The membranes were washed with TBST, reacted with electrochemiluminescence (ECL) and measured using an Imager as described before [37]. Mouse anti-glyceraldehyde 3- phosphate dehydrogenase antibody (1:10,000) was used as a loading control.

### 4.4. Computing-Based Approach for Predicting and Design of APP and Tau Peptides

Our hypothesis is that Tau binds to APP. Therefore, for each protein, putative binding proteins were searched using UNIPROT [19], a protein based search under GO molecular function section. For each protein sequence result, a BLAST (basic local alignment search tool) search was executed against the other binder proteins.

GSK3 is one of the proteins that Tau interacts with (based on the paper “Linking Aβ and Tau in Late-Onset Alzheimer’s Disease: A Dual Pathway Hypothesis”) [38]. Running a local sequence protein alignment (BLAST) between GSK3 and APP, a short sequence of ~20 amino acids was found to align. Hence, this sequence might be the starting point for peptide design to bind with Tau. The opposite analysis gave a common sequence on Tau FKB1A and CD74. Using the BLASTP program, areas in both APP and Tau protein were identified as possible candidates for the requested binding peptides. Apparently, both GSK3 and ApoE3 map to the same location on APP, and the microtubule associated protein which was found to interact with APP has a similar area on the Tau protein, hence APP might interact with Tau via this sequence [40,41]. The second Tau peptide which was selected is from the N-terminus of the protein, as this region of Tau is adjacent to phopspho-Tyr18-hTau, which has been found to accompany disease progression of Alzheimer’s disease and Tauopathy [22]. 

### 4.5. Crosslinking of APP and Tau Proteins to Identify and Confirm Candidate Peptides for In Vitro/In Vivo Experiments

A mixture of APP and Tau proteins was treated with the crosslinker BS3-H12/D12. A mixture of 1 µg/10 µL of APP (575 nM final conc.) and 0.5 µg/10 uL of Tau (545 nM final conc.) was kept overnight at 4 °C. The next day 1 µL of BS3 (50µM final conc.) was added to the mixture for 1h incubation at 37 °C. The reaction was stopped by adding 1 µL of 2M Tris, pH 8.4. 

A sample of the reaction was checked by Western blot for verification of APP and Tau binding and the rest of the material was processed for LC-MS/MS. BS3 is a linear molecule which forms a covalent bridge, a crosslink between two lysine side chains. Only lysine residues that are close to each other can be crosslinked since the length of the crosslinker is about 30Å. The APP–Tau complex have two lysine residues, one on each protein, which are close enough to each other and therefore can be crosslinked. The lysine residues which were crosslinked were identified by performing LC-MS/MS and using version 2.1.1 of xQuest [42,43]. Most of the identified crosslinks were between lysine residues on the same protein. Only one crosslink was identified between APP and Tau.

### 4.6. Synthetic Peptides

Four synthetic peptides, two from APP protein (APP1 and APP2) and two from Tau protein (Tau1 and Tau2) were prepared by GL Biochem Ltd. (Shanghai, China). Custom-made materials can be shared upon reasonable request. APP 1: HFQKAKERLEAKHRERMSQVMRE; APP 2: ATVIVITLVMLKKKQYTS; Tau1: GLGDRKDQGGYTMHQD; Tau2: KPGGGQVEVKSEKLDFK

### 4.7. In Vitro Tests

#### Inhibition of Tau and APP Proteins Binding by Peptides APP1, APP2, Tau1, Tau2 and their Combinations

An enzyme-linked immunosorbent assay (ELISA) plate (96 wells) was coated with Tau protein 1µg/ml in bicarbonate PH = 8.2 50 µL/well, then incubated overnight at 4 °C. Peptides (1 µg/mL PBF) and their combinations (0.5 + 0.5 µg/ml PBF) were also incubated overnight at 4 °C in PBS 1 µg/ml. The next day, the plate was washed 3× with PBS and blocked with 3% BSA/PBS for two hours at RT. The plate was washed again 3× with PBS, and peptides or their combinations at 50 µL/well were added for four hours at RT. The plate was washed again 3× with PBS, and APP protein, 1 µg/ml per 50 µL/well, was added for ON incubation at 4 °C. The next day, the plate was washed 3× with PBS, following which anti-Aβ 1-16 (1 mg/mL) was added (1:500 dilution, 50 µL/well) for two hours at RT. The plate was washed again and anti-mouse HRP antibodies (1:10,000 dilution in PBS 1% BSA) were added at 50 µL/well. Color reaction was stopped with 50 µL/well of 1M H2S04 and read at O.D. 450. 

### 4.8. Peptide Labeling with Fluorescein and Rhodamine for Visualization of Peptide Binding

The peptides APP1, APP2, Tau1 and Tau2 were labeled with fluorescein or rhodamine. The labelling was done according to the manufacturer’s instructions and methods in Cell Biology Protein labeling with fluorescent probes [44]. Five mg of each peptide was dissolved in 0.1 M sodium carbonate pH 9.0 (1 mL) and fluorescein or rhodamine-isothiocyanate (2 mg in 0.1 mL DMF or DMSO) was added. The reaction mixture was stirred at RT for 1 h and moved for overnight incubation to 4 °C in the dark. The possible remaining fluorescein or rhodamine was quenched with ammonium chloride for 2 h at room temperature.

Free and peptide bound fluorescein or rhodamine were separated by adsorption chromatography through a Pasteur pipette column packed with Porapak Q50-80 mesh, which binds the chromophores efficiently, allowing the peptide bound fluorescein or rhodamine to pass through the column by a small retardation to the unlabeled peptide, which comes out first.

Each of the four labelled peptides was blotted on a nitrocellulose membrane applying similar concentrations and labels to each sample. The following mixture of the labeled APP and Tau peptides: APP1F + Tau1R, App1F + Tau2R, APP2F + Tau1R, APP2F + Tau2R, (fluorescein green F, rhodamine red R) were also applied to the nitrocellulose membrane. The expectation was that only actual binding between the green and red labelled peptides will yield a yellow color.

### 4.9. Histological Staining and Quantitation of Amyloid 

Mice were sacrificed (transcardially punctured, and saline-perfused) at the end of the experiment as approved by IACUC (# 34030217-3 29.11.17-29.11.19). The right brain hemisphere of each mouse was stored at −70 °C to quantify Aβ levels. The left hemispheres were fixed (4% paraformaldehyde) and prepared for paraffin blocks, which were cut into six micron sagittal sections and used for histological staining and examination. Sagittal brain sections were stained with Congo red dye, or 6E10 Aβ antibodies as described [45]. Staining was also performed with 6E10 A-Beta antibodies. Quantification analysis of amyloid depositions was performed blindly using Imaging Research software from the National Institutes of Health in an unbiased stereological approach.

### 4.10. Analysis of Amyloid Peptide from Brain Homogenate Samples

The right hemisphere of each mouse in each treatment group was homogenized with PBS containing protease inhibitors and centrifuged at 40,000× *g* for 40 min. The supernatant-containing soluble **Aβ** was aliquoted and stored at −70 °C. The levels of Aβ (1–42) in the brain samples were assessed by an ELISA kit (Mercury EZHS42, Billerica, MA 01821, USA) for high sensitivity human amyloid beta-42 [46], *n* = 5 mice/group. 

All in vitro tests were conducted during day-time. 

### 4.11. In Vivo Animal Treatment and Behavior Test

#### 4.11.1. Outline of Experimental Process

The in vivo research design employed in the study is illustrated in Figure 3.

#### 4.11.2. Animal Treatment and Behaviour Test

Mice used were double Tg 5xFADXTau (FT) yielded by a cross of heterozygous 5xFAD Tg mice (Tg 6799) with heterozygous DM hTau Tg, as well as 5xFAD mice. As controls, non-Tg littermates were used. No predetermined sample calculation was performed and no exclusion criteria was predetermined. A total number of 39 mice were used in the in vivo studies. Mice were arbitrarily chosen, but no official randomization method was used. Mice were randomly assigned to experimental treatment groups of peptide-treatment or PBS-treated controls (3–9 animals each). In total, 16 male and 6 female mice were used in the in vivo studies of FT mice. No behavioral differences were observed between the sexes when tested in the Y-maze experiment, in terms of response to treatment. Treatment, in three different experiments, started at either 3 or 6 months old mice. In vivo experiments with 5xFAD were only done starting at 3 months of age, with a total of 12 mice. Five non-transgenic mice were used as the control. No mice were excluded from the experiment. A mixture of APP1 peptide 5 µg/5 uL and Tau1 peptide 5 µg/5 uL was administered nasally every second day for 4–5 months. In the control group, PBS was administered similarly. No anesthetics/analgesics were used as neither treatment nor behavioral testing caused discomfort to the mice. The mice were checked three times a week, and no mortality or weight loss occurred. By the end of the experiment, mice were euthanized by means of CO_2_ as approved by the ethical protocol and their brains excised. One hemisphere was prepared for histology and the other was frozen in −70 °C for processing Aβ 1-42 content.

The Y-maze test [47] consists of two trials, first “training” and second “retention”, separated by an inter-trial interval (ITI). Each arm of the Y-maze was equipped with a guillotine door that could be operated manually. The three identical arms were randomly designated as follows: the “start” (steam) arm, in which the mouse began to explore the maze (always open); the “Novel” arm, which was closed off during the first trial, but open in the second one; and the “other” arm (always open). The first (training) trial lasted five minutes and allowed the mouse to explore only two arms (“start” and “other”) of the maze. Access to the third (“Novel”) arm was blocked. The second trial (retention) was conducted after a two minute ITL. During this three minute trial, all three arms were accessible. The mouse was returned to the same starting arm and was allowed to explore all three arms. Retention was scored as a preferential index to the “Novel” arm, which was calculated as follows: time spent in the “Novel” arm minus the time spent in the old arm divided by the sum of time spent in both arms [48]. 

Open field test assessments were performed in a dark gray circular arena (diameter 56 cm) under dim illumination (20 lux). The mice were placed in the arena for five minutes. Locomotion in the arena was quantified using the Noldus video tracking software (MediaRecorder and Ethovision).

All behavior testing was done in a blinded form without knowing which experimental group was being tested. Analysis of results was also done in a blinded form, by a different person to the one who performed the testing.

### 4.12. Statistical Analysis

The in vivo data were analyzed by SPSS software (version 23, IBM, Armonk, NY, USA), and Statistica (version 12, Statsoft). No test for outliers was conducted. The datasets were first tested for normality using the Shapiro–Wilk test. If the data were normally distributed, parametric comparisons were performed: one-sample or independent samples t-tests and one-way analysis of variance (ANOVA) followed by the relevant post hoc comparisons. When the data deviated significantly from normality, non-parametric tests were applied: Mann–Whiteny U-test or Kruskal–Wallis one-way ANOVA followed by Dunn’s pairwise comparison post hoc analysis. Data are presented as individual data points in a box-plot. A probability value (*p*) of less than 0.05 was considered significant.

## 5. Conclusions

Our major conclusion is that the two brain proteins, which are considered major components involved in AD, interact with each other via specific peptide regions. The mixture of the synthetic version of these two corresponding peptides or their complex, inhibits the binding of APP and Tau proteins. Biochemically, in vitro assays confirmed the relevance of these candidate peptides. In vivo assessments demonstrated that the hypothesis regarding the role of APP and Tau protein binding, in a novel Alzheimer’s (AD) animal model, is valid. Administration of this peptide mixture prevented both cerebral plaque formation and the decline in cognitive function. These effects are attained when treatment is initiated either at an early age (three months), preventing the disease from developing, or at the age of six months, when severe cognitive functions and plaque load are already evident, leading to an improvement in the cognitive ability and reduction of plaque and soluble Aβbrain load.
